# Supplementary material for: The Role of Soluble Low-Density Lipoprotein Receptor-Related Protein-1 in Obstructive Sleep Apnoea
Source: J Clin Med. 2021 Apr 3;10(7):1494. doi: 10.3390/jcm10071494 (PMC8038392; doi:10.3390/jcm10071494)
Supplement: Supplementary file 1 [file jcm-10-01494-s001.pdf]

Journal of Clinical Medicine

Supplementary material

Table S1. Patients' characteristics among the OSA severity groups.

	Mild (n = 13)	Moderate (n = 14)	Severe (n = 19)	p
Age (years)	45 (42-61)	61 (52-67)	54 (49-63)	**< 0.001**
Males (n, %)	54	64	79	**< 0.001**
BMI (kg/m^2)	24.77 (23.67-31.25)	28.84 (25.43-34.93)	32.14 (29.63-39.93)	**< 0.001**
Hypertension (%)	46	79	74	**0.004**
Diabetes (%)	8	14	21	0.647
Dyslipidaemia (%)	46	29	21	0.471
Cardiovascular disease (%)	8	7	11	0.968
Cardiac arrythmia (%)	15	21	37	0.243
Smokers (%)	31	36	26	**0.031**
SBP (mmHg)	125.0 (120.0-138.0)	143.0 (136.5-150.0)	134.5 (131.0-141.3)	**< 0.001**
DBP (mmHg)	76.0 (70.0-80.0)	89.0 (84.0-90.0)	84.0 (79.5-89.3)	**< 0.001**
CRP (mg/l)	2.48 (0.99-3.42)	2.06 (1.39-3.71)	4.04 (2.75-5.89)	**0.002**
Glucose (mmol/l)	4.9 (4.7-5.1)	5.3 (5.0-6.6)	5.7 (4.9-7.7)	**0.004**
Cholesterol (mmol/l)	6.0 (5.4-6.4)	5.2 (4.8-6.4)	5.5 (5.1-6.1)	0.738
HDL-C (mmol/l)	1.40 (1.19-1.68)	1.26 (1.01-1.50)	1.16 (1.08-1.32)	**< 0.001**
LDL-C (mmol/l)	3.79 ± 0.94	3.60 ± 0.96	3.44 ± 0.80	0.529
Triglyceride (mmol/l)	1.47 (1.06-2.02)	1.64 (1.27-2.09)	1.84 (1.36-2.16)	**0.025**
Lipoprotein (a) (mmol/l)	0.46 (0.10-0.70)	0.17 (0.01-0.44)	0.09 (0.09-0.09)	0.965
ApoA1 (g/l)	1.48 (1.29-1.68)	1.50 (1.29-1.80)	1.38 (1.35-1.44)	0.340
ApoB (g/l)	1.23 (1.18-1.40)	1.16 (0.91-1.32)	1.29 (1.11-1.44)	0.611
TyG	8.82 ± 0.39	8.93 ± 0.51	8.98 ± 0.57	**< 0.001**
AIP	0.05 ± 0.25	0.13 ± 0.25	0.15 ± 0.25	**< 0.001**
AHI (1/h)	7.7 (6.8-10.4)	22.8 (18.4-26.3)	42.4 (34.2-59.8)	**< 0.001**
ODI (1/h)	6.5 (4.4-8.7)	21.1 (14.4-22.1)	38.3 (32.5-56.8)	**< 0.001**
SPT (min)	456.55 ± 29.98	443.60 ± 40.52	438.88 ± 55.57	0.587
TST (min)	409.55 ± 24.57	419.10 ± 25.72	419.38 ± 44.03	0.640
TST90% (%)	0.3 (0.0-0.7)	5.1 (2.8-7.6)	16.4 (6.2-40.4)	**< 0.001**
MinSatO_2 (%)	88.0 (84.0-89.0)	81.0 (75.0-83.0)	77.0 (69.5-86.0)	**< 0.001**
ESS	7 (3-9)	6 (5-8)	7 (5-10)	0.874

Data are presented as mean ± standard deviation or median (25-75% percentile). (AHI – apnoea-hypopnoea index, AIP – atherogenic index of plasma, BMI – body mass index, CRP – C-reactive protein, DBP – diastolic blood pressure, ESS – Epworth Sleepiness Scale, HDL-C – high-density lipoprotein cholesterol, LDL-C – low-density lipoprotein cholesterol, MinSatO_2 – minimal oxygen saturation, ODI – oxygen desaturation index, SBP – systolic blood pressure, SPT – sleep period time, TST – total sleep time, TST90% - total sleep time spent with oxygen saturation below 90%, TyG – Triglyceride glucose index)
